# Identification of QTLs for Stripe Rust Resistance in a Recombinant Inbred Line Population

**DOI:** 10.3390/ijms20143410

**Published:** 2019-07-11

**Authors:** Manyu Yang, Guangrong Li, Hongshen Wan, Liping Li, Jun Li, Wuyun Yang, Zongjun Pu, Zujun Yang, Ennian Yang

**Affiliations:** 1 Crop Research Institute, Sichuan Academy of Agricultural Sciences, Chengdu 610066, China; 2 School of Life Science and Technology, University of Electronic Science and Technology of China, Chengdu 610054, China; 3 Chengdu Academy of Agricultural and Forestry Sciences, Wenjiang, Chengdu 611130, China

**Keywords:** wheat, stripe rust, QTLs, SLAF-seq, chromosome translocation

## Abstract

Stripe rust, caused by *Puccinia striiformis* f. sp. *tritici* (*Pst*), is one of the most devastating fungal diseases of wheat worldwide. It is essential to discover more sources of stripe rust resistance genes for wheat breeding programs. Specific locus amplified fragment sequencing (SLAF-seq) is a powerful tool for the construction of high-density genetic maps. In this study, a set of 200 recombinant inbred lines (RILs) derived from a cross between wheat cultivars Chuanmai 42 (CH42) and Chuanmai 55 (CH55) was used to construct a high-density genetic map and to identify quantitative trait loci (QTLs) for stripe rust resistance using SLAF-seq technology. A genetic map of 2828.51 cM, including 21 linkage groups, contained 6732 single nucleotide polymorphism markers (SNP). Resistance QTLs were identified on chromosomes 1B, 2A, and 7B; *Qyr.saas-7B* was derived from CH42, whereas *Qyr.saas-1B* and *Qyr.saas-2A* were from CH55. The physical location of *Qyr.saas-1B*, which explained 6.24–34.22% of the phenotypic variation, overlapped with the resistance gene *Yr29*. *Qyr.saas-7B* accounted for up to 20.64% of the phenotypic variation. *Qyr.saas-2A*, a minor QTL, was found to be a likely new stripe rust resistance locus. A significant additive effect was observed when all three QTLs were combined. The combined resistance genes could be of value in breeding wheat for stripe rust resistance.

## 1. Introduction

Bread wheat (*Triticum aestivum*) is one of the most important food crops for mankind and the security of wheat production benefits economic development and social stability. However, wheat production in China is continually challenged by diseases, including rusts, powdery mildew, and Fusarium head blight. Stripe rust, caused by *Puccinia striiformis* f. sp. *tritici* (*Pst*), is one of the most devastating fungal diseases in many areas around the world. Beddow et al. [1] estimated that up to 88% of the world’s wheat cultivars had become susceptible since 1960 and that annual losses amounted to 5.47 million tonnes. Resistance is recognized as the most effective, economic, and environmentally safe strategy for control of stripe rust, although fungicides can also effectively control the disease, provided they are used in a timely and safe manner [2,3].

Resistance to stripe rust is generally categorized as seeding (or all-stage) resistance and adult-plant resistance (APR, including high temperature APR) according to the growth stage at which it is expressed [2,4]. Up until now, seventy-nine genes for stripe rust resistance (*Yr1* to *Yr79*) have been permanently named, but dozens of temporarily designated and hundreds of quantitative trait loci (QTL) have been reported and mapped to the wheat genome [5,6]. Among the formally designated stripe rust resistance genes, 55 confer seeding resistance and 24 genes are described as APR genes [6,7,8,9,10]. Some of these genes have been very widely deployed in agriculture by major epidemics following the emergence and increase of new virulent pathogen races. Examples of such occurrences of “boom and bust” situations in China include the use of *Yr1* from the 1950s, *Yr9* from the 1970s (overcome by Chinese yellow rust race CYR29), so-called Fan 6 resistance from the 1990s (race CYR31), and *Yr24*/*Yr26* from the 2000s (CYR34). At the time of its downfall in the late 1990s, *Yr9*, present in a 1RS·1BL translocation, was deployed in more than 80% of the released cultivars in China [11]. The lessons learnt from these events were that widespread deployment of a single highly effective resistance gene ultimately leads to failure, with detrimental effects proportional to area of wheat cultivars bearing that gene. With the aim to avoid the overuse of individual resistance genes, avoid deployment of combinations of effective resistance genes, and to use resistance, sources with reputed durability were generally applied. It is therefore necessary to find new sources of stripe rust resistance to identify the underlying genes for resistance and to convince breeders that they are worthy of use in current breeding programs.

The stripe rust in Sichuan province is the most serious foliar disease affecting wheat production. Wheat cultivars Chuanmai 42 (CH42) and Chuanmai 55 (CH55) developed by the Crop Research Institute, Sichuan Academy of Agricultural Sciences, were released in 2004 and 2009, respectively. CH42 was a synthetic wheat derivative with *YrCH42* (*=Yr24/Yr26*) [12] and maintained novel stripe rust resistance before 2010 in Sichuan [13]. As a new high-yielding and excellent quality variety, CH55 was selected from the cross SW3243/SW8688. It displayed a high level of resistance to stripe rust over a decade. However, the molecular genetic basis of CH55 for stripe rust resistance at adult plant stage has not been investigated.

Specific locus amplified fragment sequencing (SLAF-seq) is a recently developed, high-throughput strategy for large-scale single nucleotide polymorphism (SNP) discovery and genotyping, based on next generation sequencing (NGS) technology [14]. It is also cost-effective. SLAF-seq technology has been used in various species and different types of populations. For example, a high-density genetic map of cucumber (*Cucumis sativus* L.) spanning 845.87-cM with an average genetic distance of 0.45 cM was constructed for an F_2_ population [15]. Zhang et al. [16] similarly applied an SLAF-seq strategy in constructing a genetic map of 907.8 cM for a segregating *Agropyron* F_1_ population. Hu et al. [17] identified and cloned a candidate gene associated with thousand-grain weight (TGW) in wheat using DNA bulks of recombinant inbred lines (RILs). Yin et al. [18] fine-mapped the stripe rust resistance gene *YrR39* to a 17.39 Mb segment on wheat chromosome 4B using SLAF-seq combined with bulked segregant analysis (BSA) of F_2_ and BC_1_ progenies. However, SLAF-seq has not been used to construct a high-density genetic map for an entire wheat RIL population and then identify QTLs for disease resistance.

In this study, an RIL population from a cross between CH42 and CH55 were developed for QTL mapping of stripe rust resistance and the SLAF-seq for individual RILs was used to construct a high-density genetic map for identifying the QTLs from CH55 and CH42 backgrounds.

## 2. Results

### 2.1. Fluorescence In Situ Hybridization (FISH) Analysis of CH55 and CH42

**Figure 1:**
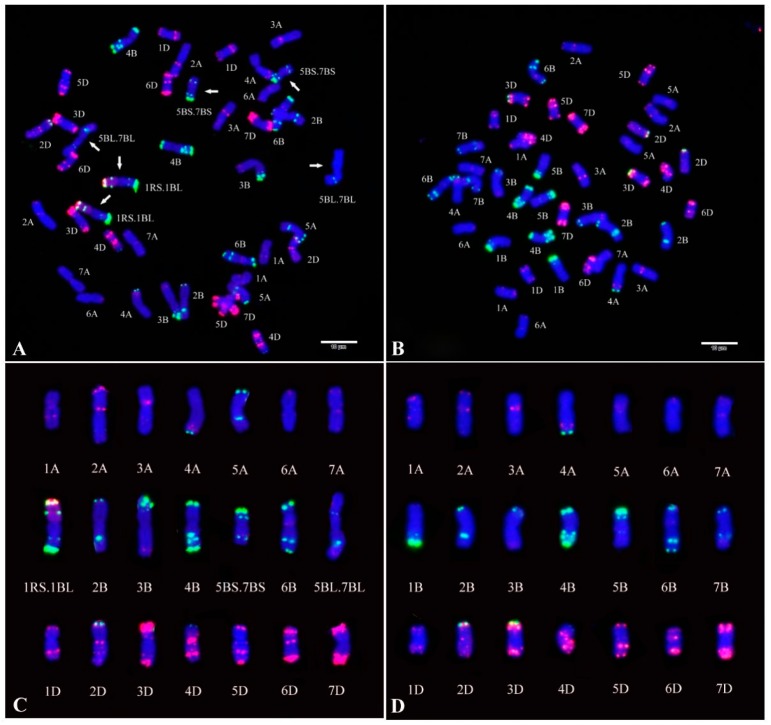
Fluorescence in situ hybridization (FISH) and karyotypic analysis of the root tip metaphase chromosomes of (**A**,**C**) CH55 and (**B**,**D**) CH42. Arrows show the translocation chromosomes. FISH was conducted using Oligo-pTa535-1 (red) and Oligo-pSc119.2-1 (green) as probes. Chromosomes were counterstained with 4’,6-diamidino-2-phenylindole (DAPI, blue). Scale bar: 10 μm.

Non-denaturing FISH (ND-FISH) with the probes Oligo-pSc119.2-1 and Oligo-pTa535-1 was conducted to reveal the chromosome—composition of the two parents, CH55 and CH42. Compared with the standard FISH karyotype of wheat and rye chromosomes indicated by Tang et al. [19], we found that CH55 carried a pair of 1RS·1BL translocation and two pairs of 5B–7B reciprocal translocation chromosomes, whereas CH42 had a normal wheat karyotype (Figure 1).

### 2.2. Phenotypic Analysis

**Figure 2:**
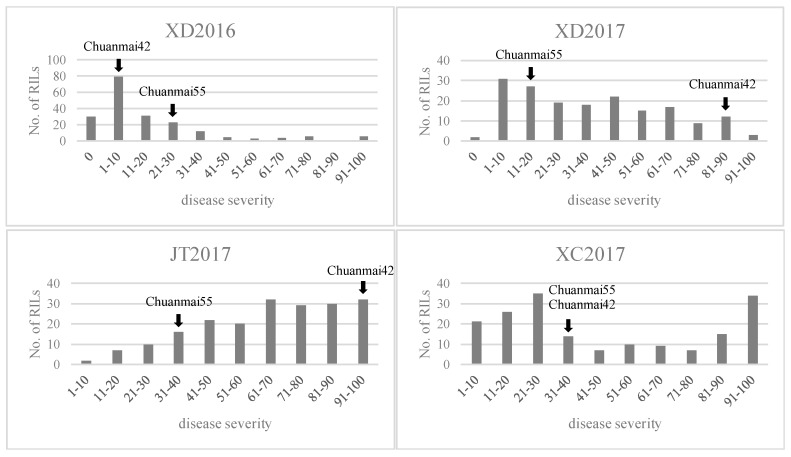
Frequency distributions of disease severities in four environments.

The frequency distributions of disease severities for stripe rust reaction at adult plant stages in each environment ranged over 0–100, 0–95, 2.5–100, and 0.5–100 at XD2016, XD2017, JT2017, and XC2017, respectively, showing continuous variation (Figure 2). The results indicated that stripe rust resistance in CH55 and CH42 was possibly controlled by multiple genes. In all four environments there was transgressive segregation in both directions (Table 1). Broad sense heritability (*H*^2^) was 0.88 (Table 1), indicating the data could be used for further QTL mapping.

### 2.3. Analysis of SLAF-Seq Data and SNP Markers

After SLAF library construction and sequencing, 354.218 Gb of data containing 1771.45 M paired-end reads were obtained; 94.82% of the bases were of high quality with Q30 (a quality score of 30 indicates a 1% chance of error, thus a 99% accuracy) and the guanine–cytosine (GC) content was 44.79% (Table 2). A total 2,825,198 SLAFs were developed. The SLAFs numbers for CH42 and CH55 were 862,053 and 863,835, and their corresponding average sequencing depths were 26.70 and 24.15, respectively. The average number of SLAFs for the RIL population was 493,537 and the average sequencing depth was 11.80 (Table 3). The range of reads in the RILs was 2,422,631 to 14,454,545.

The 1771.45 M paired-end reads, consisting of 2,825,198 SLAFs, contained 2,507,026 SNPs. After filtering 15,563 multiple mutation sites, 2,491,463 SNPs were used for subsequent analysis. Among those, the markers in which the bases were absent in the paternal or maternal parent were filtered, leaving 640,734 markers. Then, markers with average sequence depths < 4 were filtered, leaving 446,616 markers. Among the 446,616 SNP markers, 162,394 markers were polymorphic SNPs with a polymorphic rate of 36.36%. All the polymorphic SNP markers were classified into four genotypes—aa × bb, hk × hk, lm × ll, and nn × np. However, only the genotype aa × bb, consisting of 75,347 SNP markers, which accounted for 3.01% of the total SNP markers, was used for further analysis. Finally, markers with parental sequence depths of less than ten and significant segregation distortions of less than 0.01 (*p* < 0.01) were filtered and the remaining 6732 markers were used to construct the final genetic map (Table 4).

### 2.4. Genetic Map Construction and Consistency Analysis

**Figure 3:**
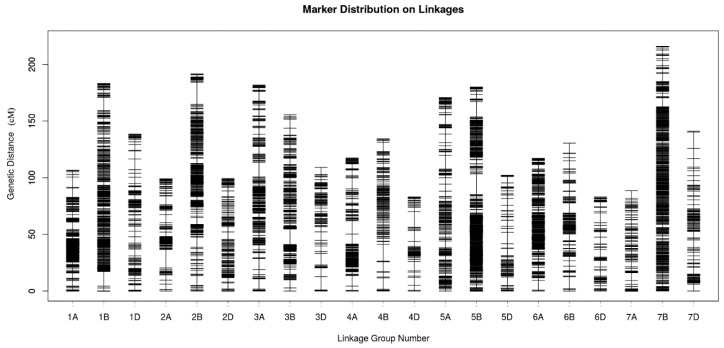
Distribution of SNP markers on individual chromosomes.

The genetic map of 21 linkage groups was 2828.51 cM, with an average marker interval of 0.42 cM. The sub-genome statistics are provided in Table 5. The largest chromosome was chromosome 7B and the shortest was chromosome 6D. The largest gap in the map was 19.46 cM, which was located on chromosome 2B. The largest proportion of gaps less than 5 cM was 99.77% for chromosome 6A, whereas the smallest proportion of gaps, 93.85%, was for chromosome 4D (Figure 3).

The consistency analysis of the SNP loci between the genetic map and the physical map is shown in Table 6. Among all 21 linkage groups, there were 10 chromosomes with Spearman coefficients between 0.8 and 0.9, including 1A, 2D, 3A, 4B, 4D, 5B, 5D, 6A, 6D, and 7A. The Spearman coefficients for the other 11 chromosomes were between 0.9 and 1.0. The results indicated that the locations of most SNP loci on the genetic map were consistent with their corresponding physical locations in the Chinese Spring genome.

### 2.5. QTL Mapping of Stripe Rust Resistance of the RILs

**Figure 4:**
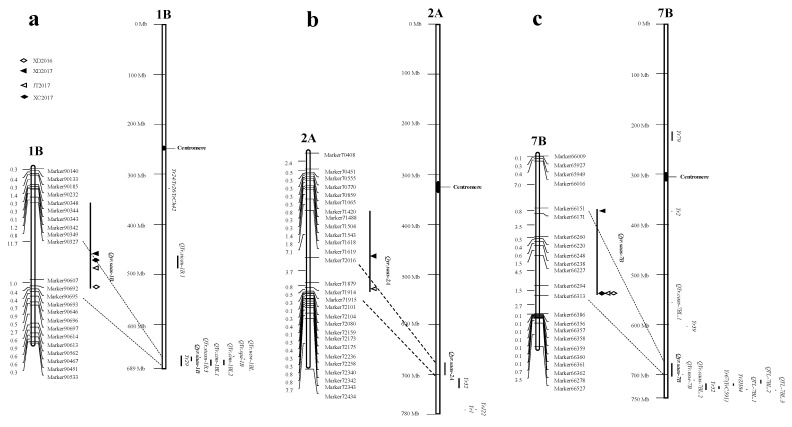
Locations of detected QTLs and comparison with physical positions of other *Yr* genes/QTLs. (**a**) The genetic and physical location of *Qyr.saas-1B*; (**b**) the genetic and physical location of *Qyr.saas-2A*; (**c**) the genetic and physical location of *Qyr.saas-7B*. 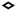 represents field trials in 2015–2016 growing seasons at Xindu (XD2016); 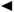 represents field trials in 2016–2017 growing seasons at Xindu (XD2017); 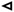 represents field trials in 2016–2017 growing seasons at Jitian (JT2017); 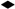 represents field trials in 2016–2017 growing seasons at Xichong (XC2017).

Three QTLs were identified for stripe rust resistance using inclusive composite interval mapping (ICIM) with logarithm of odds (LOD) scores of 3.05–21.34, explaining 3.27–34.22% of the phenotypic variation (Figure 4, Table 7). These QTLs were identified on chromosomes 1B, 2A, and 7B and were temporarily designated *Qyr.saas-1B*, *Qyr.saas-2A*, and *Qyr.saas-7B*. *Qyr.saas-7B* was derived from CH42, whereas the other two QTLs were from CH55.

*Qyr.saas-1B* and *Qyr.saas-7B* were detected in all four environments, explaining 6.24%–34.22% and 3.27–20.64% of the phenotypic variation, respectively. *Qyr.saas-1B* was located in a 13.05 cM interval flanked by markers 90327 from XD2017 and 90695 from XD2016 and *Qyr.saas-7B* was located in a 13.16 cM interval flanked by markers 66151 from XD2017 and 66313 from XD2016. *Qyr.saas-2A,* with a smaller effect, was detected in only two environments and was located in a 12.12 cM region flanked by markers 71619 from XD2017and 71915 from JT2017, explaining 3.77–5.29% of the phenotypic variation.

### 2.6. Additive Effects of QTLs

The RILs were divided into 8 groups based on the genotypes of the closest markers for each of the three QTLs (Table 8). Significant additive effects were found in RILs with two or more resistance QTLs. *Qyr.saas-1B* and *Qyr.saas-7B* significantly reduced disease severity in all four environments when present alone. When present together in XC2017, they acted in an additive fashion and conferred lower severity than either QTL alone. A similar additive effect occurred in JT2017 in combinations of *Qyr.saas-1B* and *Qyr.saas-2A*. The lowest severities occurred when all three QTLs were combined in XD2016; the disease severities of many RILs approached immunity. Extremely low disease severity scores also occurred in XD2017 and XC2017, but were not apparent in JT2017.

## 3. Discussion

In order to determine the relationship between the three QTLs identified in the present study and other *Yr* genes and QTLs reported previously, we compared their physical locations by basic local alignment search tool (BLAST) analysis of the International Wheat Genome Sequencing Consortium (IWGSC) RefSeq v1.0 genome, which was shown in Figure 4 and Table 9.

*Qyr.saas-1B*, contributed by CH55, was significantly associated with resistance to stripe rust in all environments. It was physically located between 664.08 Mbp and 673.64 Mbp in the distal region of chromosome 1BL (Table 9, Table S1, Figure 4a). This region is rich in stripe rust resistance genes and QTLs, such as *Yr21* [20], *Yr24*/*Yr26* [21], and *Yr29* [22,23]. The physical interval of *Qyr.saas-1B* overlapped with *Yr29*, *QYr.cim-1BL1* [24], *QYr.cim-1BL2* [25], *QYr.spa-1B* [26], *QYr.ucw-1BL* [23,27], and *QYr.sicau-1B.3* [28]. We concluded that *Qyr.saas-1B* was most likely *Yr29*. However, more work is needed to conclude that *Qyr.saas-1B* is *Yr29*. The *Yr29* is an APR gene first reported in cultivar Pavon 76 [29], but has since been identified in many different genetic backgrounds, including Pastor [30], Francolin#1 [24], and Klein Chajá [27]. In the present study, CH55 showed high resistance to stripe rust in all four environments with low disease severities of 15–40, with *Qyr.saas-1B* explaining 6.24%–34.22% of the phenotypic variation. This indicates that *Qyr.saas-1B* is relatively effective in Sichuan. Moreover, extremely low disease severity scores occurred when *Qyr.saas-1B* was combined with the other two QTLs with rather positive additive effects also being detected (Table 8). Therefore, the *Qyr.saas-1B* is considered to be a valuable component of resistance for use in Sichuan breeding programs combined with other genes.

*Qyr.saas-7B,* derived from CH42, also had consistent QTLs across the environments; it was physically located between 678.64 Mbp and 706.81 Mbp in the distal region of chromosome 7BL (Table 9, Table S1, Figure 4c). Several permanently- and temporarily-named stripe rust resistance genes have been mapped to chromosome 7BL (Table 9), including *Yr2* [31], *Yr39* [32], *Yr52* [33], *Yr59* [34], *Yr67* (*YrC591*) [35], *Yr79* [6], *YrZH84* [36], and *YrMY37* [7]. None of the physical intervals for these genes overlapped with *Qyr.saas-7B* (Table 9, Figure 4c). A number of QTLs were also mapped to chromosome 7BL (Table 9), including *QTL-7B.1* [37], *QTL-7B.2* [38], *QTL-7B.3* [39], *QYr.nsw-7B* [40], *QYr.caas-7BL.1,* and *QYr.caas*-*7BL.2* [33]. The physical interval of *QYr.nsw-7B* from Tiritea [40] overlapped with *Qyr.saas-7B* (Table 9, Figure 4c), suggesting they could be the same locus. Similar to the genetic variation across environments, both *QYr.nsw-7B* [40] and the present *Qyr.saas-7B* could be important as a component of multiple-gene resistance to stripe rust. Previous study has located a stripe rust resistance gene *YrCH42* on the 1B chromosome of CH42 [12], but the QTL of *Qyr.saas-7B* from CH42 was not detected as it was in this study. It is possible that the stripe rust resistance of *YrCH42* was overcome with the occurrence of *Pst* race CYR34, which was inoculated in this study [13]. There is another possibility that the present study used the high-throughput SLAF markers to screen an entire wheat RIL population between CH45 and CH42, which is higher resolution than the previous study for CH42 by SSR-PCR assay [12].

A minor QTL from CH55 was identified on chromosome 2AL. The *Qyr.saas-2A* was physically located between 677.90 Mbp and 701.74 Mbp in chromosome 2AL (Table 9, Table S1, Figure 4b). *Yr1* [41], *Yr32* [42], and *YrJ22* [43] were mapped to 2AL, but these were genes of large effect. *Qyr.saas-2A* was likely to be a new stripe rust resistance locus based on its different physical location (Table 9, Figure 4b). This QTL was detected only in XD2017 and JT2017, which explained 3.77% and 5.29% of the phenotypic variation, respectively (Table 7). The effect of *Qyr.saas-2A* was much smaller than that of *Qyr.saas-1B* and *Qyr.saas-7B*. However, a significant additive effect in *Qyr.saas-2A* was observed. Singh et al. [44] indicated that an adequate level of slow rusting resistance could be achieved by the additive/complementary effects of three to five genes. This has been supported by many reports, including those of Yang et al. [45], Lan et al. [24], and Rosewarne et al. [46]. Similarly, the disease severities of RILs approached immunity when *Qyr.saas-2A* was combined with two other QTLs, *Qyr.saas-1B* and *Qyr.saas-7B,* in XD2016. There is repeated evidence that an effective and stable level of adult plant stripe rust resistance can be achieved by using combinations of genes that individually confer relatively small effects. Therefore, although the effect of *Qyr.saas-2A* was small, it provided enhancement effects and therefore could be useful in Sichuan wheat breeding for multiple gene resistance to stripe rust.

Based on the studies of chromosome composition of CH55 revealed by FISH analysis (Figure 1), we found that CH55 contained both 1RS·1BL and 5B-7B reciprocal translocation chromosomes. The 1RS·1BL translocation is still widely used in wheat breeding because of the superior genes for grain yield and stress tolerances in 1RS [47]. In the present study, the 1BL arm of 1RS·1BL in CH55 carried the stripe rust resistance QTL *Qyr.saas-1B*. According to a previous study, the alien chromatin suppresses the recombination between normal and translocated chromosomes [48]. Therefore, the selection of 1RS·1BL accumulates excellent agronomic characteristics and resistance with high frequency in breeding practice. Moreover, the 5B–7B reciprocal translocation is possibly of French origin according to the genealogy of CH55. It was found that the stripe rust resistance QTL *QYr.ufs-5B* was located on 5BS in the 5B–7B reciprocal translocation [49], which requires further validation by *Pst* races in different environments for CH55.

## 4. Materials and Methods

### 4.1. Plant Materials and Field Trials

A set of 200 F_6_ RILs developed from cross CH42/CH55 and parents were used to evaluate stripe rust responses in multiple environments. CH42 and CH55, developed by Crop Research Institute, Sichuan Academy of Agricultural Sciences, were released in 2004 and 2009, respectively. CH42 is a synthetic wheat derivative produced from cross SynCD768/SW3243//Chuan 6415 and CH55 was selected from the cross SW3243/SW8688. Chuanyu12, developed by Chengdu Institute of Biology of Chinese Academy of Sciences, is highly susceptible to the currently prevalent *Pst* races in Sichuan province and was used as spreader.

Field trials were conducted at the Xindu (Sichuan Province) research station in the 2015–2016 (XD2016) and 2016–2017 (XD2017) growing seasons and also at Jitian and Xichong (Sichuan Province) in 2016–2017 (JT2017, XC2017). Field trials were conducted in randomized complete blocks with two replications. Plots were sown as single 1 m rows, 25 cm apart, and about 30 seeds were sown in each row. Every 20th row was planted with the susceptible cultivar Chuanyu 12 as a spreader to produce an inoculum and as a control. The surrounding spreaders were inoculated with a mixture of currently prevalent *Pst* races, including CYR33, CYR34(V26), and G22-14. Adult-plant disease severities were visually recorded as 0–100% according to the modified Cobb Scale of Peterson et al. [50] when severities on Chuanyu 12 reached 90–100%, usually 1–2 weeks post-anthesis.

### 4.2. Broadsense Heritability

Phenotypic variance per plot in multi-trials can be written as $\sigma_{P}^{2}$ = $\sigma_{G}^{2}$ + $\sigma_{GE}^{2}$ + $\sigma_{\varepsilon }^{2}$, where $\sigma_{G}^{2}$ is the genetic variance, $\sigma_{GE}^{2}$ is the variance for genotype-environment interaction, and $\sigma_{\varepsilon }^{2}$ is the residual variation. Broad sense heritability in on an individual plot basis was calculated with the formula *H*^2^ $=\;\frac{\sigma_{G}^{2}}{\sigma_{G}^{2}+\sigma_{GE}^{2}+\sigma_{\varepsilon }^{2}}$.

### 4.3. DNA Extractions

Young leaf tissues of 1 plant per parent and RILs were sampled in 2016, stored at −80 °C, and used for DNA extraction. The genomic DNA from each genotype was extracted using the cetyltrimethylammonium ammonium bromide (CTAB) method.

### 4.4. SLAF Library Construction and High-Throughput Sequencing

In this project, we used the wheat reference genome version IWGSC1.0 downloaded from ftp://ftp.ensemblgenomes.org/pub/plants/release-30/fasta/triticum_aestivum/dna/. SLAF-seq was used to genotype the 200 RILs and parents using the procedure designed by Sun et al. [14], with minor modifications. DNA was digested into 464–484 bp fragments using restriction enzyme RsaI. The digested fragments were modified by adding nucleotide A and Dual-index sequencing adapters were ligated to the A-tailed DNA, which was amplified and purified to the target fragments. The purified fragments were sequenced on an Illumina HiSeq^TM^ platform.

### 4.5. Analysis of SLAF-Seq Data and Genetic Map Construction

SLAF marker identification and genotyping were performed following Sun et al. [14]. SNP loci in each SLAF locus were detected using the genome analysis toolkit (GATK) software. A genetic map was constructed for filtered markers using HighMap software and referring to the procedure detailed by Zhang et al. [51]. Spearman coefficients were used to analyze the consistency between the genetic and physical maps.

### 4.6. QTL Analysis

QTL IciMapping V4.0 [52,53] was used to identify QTLs by ICIM. A LOD score of 3.0 was used as a threshold for the declaration of linkage and the Kosambi mapping function was used to convert recombination frequencies into map distances. If a QTL was detected in a single environment, it was discarded. A positive additive effect indicated that the favorable allele was from the CH55 parent, whereas a negative additive effect indicated that the favorable allele was from CH42.

### 4.7. FISH Analysis

Root-tip metaphase chromosomes of wheat cultivars CH42 and CH55 were prepared, as described by Han et al. [54]. The probes Oligo-pSc119.2-1 and Oligo-pTa535-1 were used to detect structural variations in this study [19]. The Oligo-pSc119.2-1 was 5′-end-labeled with 6-carboxyfluorescein (6-FAM, green) and the Oligo-pTa535-1 was 5′-end-labeled with 6-carboxytetramethylrhodamine (TAMRA, red). The detailed process of FISH was performed following Tang et al. [19].

## 5. Conclusions

This study constructed a high-density genetic map and identified three QTLs for stripe rust resistance using the CH42/CH55 RILs via SLAF-seq technology. The genetic map of 21 linkage groups was 2828.51 cM, with an average marker interval of 0.42 cM. *Qyr.saas-7B* was derived from CH42, whereas *Qyr.saas-1B* and *Qyr.saas-2A* were from CH55. *Qyr.saas-2A* was likely to be a new stripe rust resistance locus. A significant additive effect was observed when all three QTL were combined. The combined resistance genes could be of value in breeding wheat for stripe rust resistance.

## Figures and Tables

**Table 1:** Stripe rust statistics for four environments.

Environment	Parent Mean		RIL Population Mean			*H* ^2^
	CH55	CH42	Min	Max	Mean	
XD2016	30	10	0	100	20.96	0.88
XD2017	15	85	0	95	39.54	
JT2017	35	95	2.5	100	66.91	
XC2017	40	40	0.5	100	48.41	

**Table 2:** Statistical data from sequencing.

Sample	Total Reads	Total Bases	Q30 Percentage (%)	GC (%)
CH42	31,435,118	6,285,898,686	97.82	44.96
CH55	33,764,850	6,752,071,114	97.96	44.85
Offspring	8,531,292	1,705,900,571	94.82	44.79
Total	1,771,458,398	354,218,084,068	94.82	44.79

The data of offspring in the table are averages.

**Table 3:** The data statistics of specific locus amplified frangments (SLAFs).

Sample	SLAFs Number	Total Depth	Average Depth
CH42	862,053	23,017,201	26.7
CH55	863,835	20,864,394	24.15
Offspring	493,537	5,744,268	11.8

**Table 4:** Steps in marker filtering for map construction.

Filtering Step	Number of SNPs
All reads	1771.45 M
SLAFs in the reads	2,825,198
SNPs in the SLAFs	2,507,026
Filtered multiple mutation sites	2,491,463
SNP without base deletion in the paternal or maternal parents	640,734
Sequence depth of SNPs > 4	446,616
Polymorphic SNPs	162,394
SNPs of genotype AA × BB	75,347
SNPs with parental sequence depth >10 and non-significant segregation distortion (*p* > 0.01)	6732

**Table 5:** Detailed information for the high-density genetic map.

Genome	Chromosome	Marker Number	Total Distance	Average Distance	Gap < 5 cM (%)	Largest Gap
A	1A	823	106.63	0.13	99.64	9.07
	2A	350	99.09	0.28	99.14	16.36
	3A	290	181.75	0.63	97.58	12.77
	4A	321	117.36	0.37	98.75	11.72
	5A	242	170.73	0.71	97.10	12.31
	6A	443	117.14	0.26	99.77	8.64
	7A	142	88.56	0.62	98.58	6.85
	Subtotal	2611	881.26	0.34	--	--
B	1B	491	183.21	0.37	99.59	12.92
	2B	474	191.60	0.40	99.15	19.46
	3B	565	155.65	0.28	99.11	13.94
	4B	136	134.38	0.99	97.04	14.91
	5B	762	180.01	0.24	99.74	18.20
	6B	322	130.55	0.41	97.82	10.64
	7B	557	215.86	0.39	99.64	7.98
	Subtotal	3307	1191.26	0.36	--	--
D	1D	159	138.40	0.87	96.20	9.35
	2D	151	99.31	0.66	98.00	6.60
	3D	104	109.02	1.05	95.15	11.72
	4D	66	83.00	1.26	93.85	13.77
	5D	103	102.24	0.99	94.12	10.07
	6D	83	82.98	1.00	93.90	8.52
	7D	148	141.04	0.95	95.24	14.52
	Subtotal	814	755.99	0.93	--	--
Total		6732	2828.51	0.42	--	--

**Table 6:** Spearman coefficient for each linkage group and physical map.

Chromosome	Spearman ^1^
1A	0.886
2A	0.999
3A	0.821
4A	1.000
5A	0.975
6A	0.807
7A	0.875
1B	0.961
2B	0.967
3B	1.000
4B	0.846
5B	0.873
6B	0.989
7B	0.939
1D	0.970
2D	0.857
3D	0.989
4D	0.805
5D	0.892
6D	0.812
7D	1.000

^1^ A value of 1 indicates perfect collinearity.

**Table 7:** QTLs for stripe rust resistance in the CH42/CH55 RIL population tested in four environments.

QTL.	Trial	Position	Left Marker	Right Marker	LOD	PVE (%)	Add ^a^
*Qyr.saas-1B*	XD2016	172	Marker90692	Marker90695	3.28	6.24	6.07
	XD2017	167	Marker90327	Marker90607	21.34	34.22	17.34
	JT2017	169	Marker90327	Marker90607	12.47	22.07	11.73
	XC2017	168	Marker90327	Marker90607	12.00	22.19	16.88
*Qyr.saas-2A*	XD2017	67	Marker71619	Marker72016	3.42	3.77	5.75
	JT2017	72	Marker71914	Marker71915	3.74	5.29	5.73
*Qyr.saas-7B*	XD2016	205	Marker66294	Marker66313	8.29	16.82	−9.94
	XD2017	192	Marker66151	Marker66171	3.05	3.27	−5.35
	JT2017	205	Marker66294	Marker66313	3.22	4.45	−5.28
	XC2017	205	Marker66294	Marker66313	13.19	20.64	−16.42

^a^ A positive additive effect indicates that the favorable alleles came from CH55; a negative additive effect indicates that the favorable allele was from CH42. XD, JT, and XC, which are denoted Xindu, Jitian and Xichong, respectively.

**Table 8:** Mean disease severities of CH42 × CH55 RILs with different genotypic combinations.

QTLs			No. of RILs with Corresponding QTL or QTL Combination	Mean Disease Severity			
1BL	2AL	7BL		JT2017	XD2016	XD2017	XC2017
+	+	+	23	38.22 a	3.95 a	15.78 a	12.09 a
+	+	-	14	52.32 ab	18.93 b	24.64 ab	45.89 b
+	-	+	17	59.74 bc	7.71 ab	23.62 ab	17.59 a
-	+	+	14	68.93 cd	11.79 ab	42.18 cd	37.68 b
+	-	-	12	71.25 cd	16.25 ab	33.54 bc	45.63 b
-	-	+	21	72.62 cd	14.52 ab	56.90 de	46.55 b
-	+	-	19	77.50 de	44.21 c	54.21 d	80.13 c
-	-	-	14	90.00 e	43.93 c	70.36 e	86.96 c

The same letter within a column indicates no significant difference at *p* > 0.05. “+” means containing the corresponding QTL while “-“ means no QTL.

**Table 9:** Comparison of physical positions of reported *Yr* genes (QTLs) with *Qyr.saas-1B*, *Qyr.saas-7B*, and *Qyr.saas-2A*.

Chromosome Arm	Genes/QTLs	Left Marker	Right Marker	Left Physical Position	Right Physical Position	Reference
1BL	*Qyr.saas-1B*	Marker90327	Marker90695	664079816	673644678	This study
	*Yr21*	M1(Pto kin2/S2)	M2(Pto kin3/PtoFen-S)	None	None	[20]
	*Yr24/Yr26/YrCh42*	WRS467	CM1641	328642215	328642801	[21]
	*Yr29*	Xgwm44	Xgwm140	662195228	684861809	[22,23]
	*QYr.sicau-1B.1*	Xwmc156	Xwmc216	461685422	487427087	[28]
	*QYr.sicau-1B.3*	AX-108726041	AX-111056129	667604743	667641255	[28]
	*QYr.cim-1BL1*	Xgwm259	Xgwm140	672333339	684861809	[24]
	*QYr.cim-1BL2*	WPt-1770	WPt-9028	671741402	681848783	[25]
	*QYr.spa-1B*	Wsnp_Ra_c53181_56932563	Wsnp_Ra_c53181_56932563	664804354	664804467	[26]
	*QYr.ucw-1BL*	IWA8581	csLV46G22	670389674	None	[23,27]
7BL	*Qyr.saas-7B*	Marker66151	Marker66313	678635912	706808017	This study
	*Yr2*	WMC364	WMC364	375022989	375023010	[31]
	*Yr39*	Xgwm131	Xgwm43	604774088	None	[32]
	*Yr52*	Xcfa2040	Xbarc182	718432553	732366237	[33]
	*Yr59*	Xbarc32	Xwmc557	723876921	728084216	[34]
	*Yr67 (YrC591)*	Xbarc32	Xbarc182	723876921	732366237	[35]
	*Yr79*	Xbarc72	Xwmc335	214059722	233160839	[6]
	*YrZH84*	Xcfa2040	Xbarc32	718432553	723876921	[36]
	*YrMY37*	Xgwm297	Xbarc267	237502276	377136685	[7]
	*QTL-7BL.1*	IWA3155	IWA3416	732651049	732651181	[37]
	*QTL-7BL.2*	Xgwm577	Xwmc166	711234115	719852469	[38]
	*QTL-7BL.3*	IWB58601	IWB58601	732651454	732651554	[39]
	*QYr.nsw-7B*	Xgwm611	Xgwm611	700632085	700632104	[40]
	*QYr.caas-7BL.1*	Xbarc176	XwPt8106	557048410	None	[33]
	*QYr.caas-7BL.2*	Xgwm577	XwPt4300	711234115	None	[33]
2AL	*Qyr.saas-2A*	Marker72016	Marker71915	677899968	701739954	This study
	*YR1*	Xgwm311	None	772967422	None	[41]
	*Yr32*	Xwmc198	Xwmc181	707741852	728609562	[42]
	*YrJ22*	Xwmc658	IWA1348	771166682	None	[43]

## Supplementary Materials

Supplementary materials can be found at https://www.mdpi.com/1422-0067/20/14/3410/s1.

## Abbreviations

Pst	*Puccinia striiformis* f. sp. *tritici*
APR	Adult-Plant Resistance
CYR	Chinese Yellow Rust
QTL	Quantitative Trait Loci
SNP	Single Nucleotide Polymorphism
CH42	Chuanmai 42
CH55	Chuanmai 55
SLAF-seq	Specific Locus Amplified Fragment Sequencing
NGS	Next Generation Sequencing
TGW	Thousand Grain Weight
RILs	Recombinant Inbred Lines
BSA	Bulked Segregant Analysis
FISH	Fluorescence In Situ Hybridization
ND-FISH	Non-denaturing FISH
DAPI	4’,6-diamidino-2-phenylindole
GC	Guanine Cytosine
ICIM	Inclusive Composite Interval Mapping
LOD	Logarithm of Odds
IWGSC	International Wheat Genome Sequencing Consortium
CTAB	cetyltrimethylammonium ammonium bromide
GATK	Genome Analysis Toolkit
